# The development of pharmacy brief intervention practice: overview of a research program

**DOI:** 10.1186/1940-0640-7-S1-A21

**Published:** 2012-10-09

**Authors:** Ranjita Dhital, Ian J Norman, Natasha S Khan, Jim McCambridge, Peter Milligan

**Affiliations:** 1 Division of Health and Social Care Research, King's College London, London, UK; 2 Institute of Pharmaceutical Science, King’s College London, London, UK; 3 London School of Hygiene & Tropical Medicine, London, UK; 4 School of Biomedical and Health Sciences, King's College London, London, UK

## 

Brief intervention (BI) delivered in pharmacies is not currently routine practice, nor is there evidence regarding its efficacy. Community pharmacies attract a large and diverse range of people to a health-care environment that is promising for BI research. Work undertaken by this research group included semi-structured interviews with pharmacy service users to obtain their views on potential pharmacy BI; a five-month cohort study with 134 participants conducted in 28 pharmacies that assessed feasibility issues; and focus-group studies with “active” and “less active” pharmacists to identify barriers and facilitators of implementing BI. Pharmacy service users were positive about participating in BI, which was found to be feasible. Training and staff support were identified as important factors influencing service delivery, and there are preliminary indications of effectiveness including an impressive reduction in overall consumption among those who completed follow-up. Pharmacist motivation has been evaluated with the Short Alcohol and Alcohol Problems Perception Questionnaire (SAAPPQ), and role adequacy and work satisfaction improved among “active” pharmacists. These outcomes are currently being used to inform the development of a randomized controlled trial of BI delivered in the pharmacy, due to begin soon. It is hoped that findings from the trial will contribute to current knowledge and promote interest in the area. Unanswered questions include how pharmacy BI can be implemented in routine practice if trial results demonstrate effectiveness, and how SBI fits with the needs of a changing pharmacy profession.

